# A spiking network model of cerebellar Purkinje cells and molecular layer interneurons exhibiting irregular firing

**DOI:** 10.3389/fncom.2014.00157

**Published:** 2014-12-01

**Authors:** William Lennon, Robert Hecht-Nielsen, Tadashi Yamazaki

**Affiliations:** ^1^Department of Electrical and Computer Engineering, University of CaliforniaSan Diego, La Jolla, CA, USA; ^2^Graduate School of Informatics and Engineering, The University of Electro-CommunicationsChofu, Japan

**Keywords:** molecular layer interneurons, Purkinje cells, cerebellum, irregular firing, inter-spike interval

## Abstract

While the anatomy of the cerebellar microcircuit is well-studied, how it implements cerebellar function is not understood. A number of models have been proposed to describe this mechanism but few emphasize the role of the vast network Purkinje cells (PKJs) form with the molecular layer interneurons (MLIs)—the stellate and basket cells. We propose a model of the MLI-PKJ network composed of simple spiking neurons incorporating the major anatomical and physiological features. In computer simulations, the model reproduces the irregular firing patterns observed in PKJs and MLIs *in vitro* and a shift toward faster, more regular firing patterns when inhibitory synaptic currents are blocked. In the model, the time between PKJ spikes is shown to be proportional to the amount of feedforward inhibition from an MLI on average. The two key elements of the model are: (1) spontaneously active PKJs and MLIs due to an endogenous depolarizing current, and (2) adherence to known anatomical connectivity along a parasagittal strip of cerebellar cortex. We propose this model to extend previous spiking network models of the cerebellum and for further computational investigation into the role of irregular firing and MLIs in cerebellar learning and function.

## Introduction

The cerebellum is thought to be involved in producing smooth and coordinated movements which are both spatially and temporally precise. How the cerebellum achieves this is not understood. One approach to elucidate this mechanism is to construct a model from known anatomy and physiology to explain how the constituent neurons compute the function implemented by the cerebellum. Numerous theoretical and computational models have been proposed (Grossberg, 1969; Marr, 1969; Albus, 1971; Fujita, 1982; Medina et al., 2000; Dean et al., 2010; Yamazaki and Nagao, 2012), however few of these models emphasize the functional role of the molecular layer interneurons (MLIs). Typically, these inhibitory interneurons are described as providing “global inhibition” or “sculpting” the overall response of the Purkinje cells (PKJs); however, recent experimental evidence questions this hypothesis (Bower, 2010; Jorntell et al., 2010). We seek to understand the role of the MLIs in concert with the PKJs which they form a vast network with by means of computational modeling.

A key feature of the network of MLIs and PKJs is that these neurons fire spontaneously in absence of excitatory synaptic input (Hausser and Clark, 1997; Raman and Bean, 1997). When inhibitory synaptic currents are blocked *in vitro*, MLIs and PKJs fire regularly (Hausser and Clark, 1997). In the presence of inhibitory synaptic currents, they exhibit relatively irregular firing. Understanding how PKJ spontaneous activity is modified to control their targets in the deep cerebellar nuclei and vestibular nuclei (DCN/VN) is central to understanding the operation of the cerebellar cortex. In conditioned eye blink response (CER) learning, PKJs learn to make an appropriately timed pause in firing in response to a conditioned stimulus, which in turn disinhibits their DCN targets and elicits an eye blink (Jirenhed et al., 2007). Since PKJs are spontaneously active and blocking excitatory synaptic inputs to PKJs only modestly decreases the spontaneous PKJ activity *in vivo* (Cerminara and Rawson, 2004) and *in vitro* (Hausser and Clark, 1997), a decrease in efficacy at parallel fiber (PF) to PKJ synapses is insufficient to explain the learned pause in PKJ activity. Feedforward inhibition provided by MLIs may be one mechanism to produce this pause. Furthermore, using an optogenetic technique to increase the firing rates of a target population of MLIs in awake mice, movements can be elicited and kinematics controlled by varying the photostimulation parameters (Heiney et al., 2014). Finally, in mutant mice lacking PKJ gamma-aminobutyric acid A (GABA_A_) receptors, effectively removing MLI feedforward inhibition, motor learning deficits are observed (Wulff et al., 2009). The accumulating evidence points to a greater functional role for MLIs than previous theories suggest.

In this study we construct a spiking network model of spontaneously active MLIs and PKJs composed of leaky integrate-and-fire neuron models connected according to known anatomy. We show that despite using simple neuron models, this network reproduces the irregular ISIs observed in PKJs and MLIs *in vitro*. We further show that the relative contribution of MLI → MLI feedback inhibition to produce irregular firing in MLIs is greater than the PKJ → MLI feedback inhibition contribution. Finally, this model provides a substrate for additional experiments investigating the functional role of irregular firing patterns and MLIs in cerebellar learning and function.

## Materials and methods

### Network model

**Figure 1:**
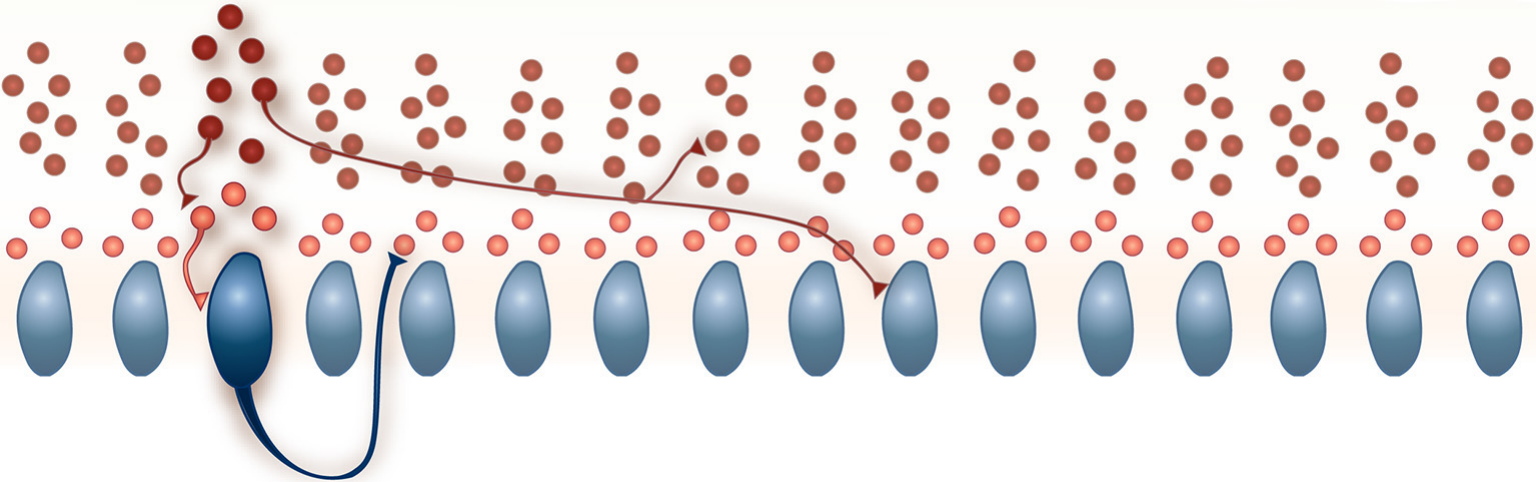
**A schematic of the model network**. 160 MLIs (red) and 16 PKJs (blue) were simulated. Each PKJ has 10 corresponding MLIs that are closest to it along the long axis. Of the 10 closest, three are eligible to receive PKJ recurrent collaterals (shown in a lighter shade of red) and seven are ineligible to receive PKJ recurrent collaterals (shown in a darker shade of red). Examples of allowed connections are shown. All synapses are inhibitory. Further details on the network connectivity are described in the Methods Section.

The network is composed of PKJs and MLIs and is modeled after a 1 mm × 32 μm microzone of the cerebellar cortex with the long axis extending parasagittally. In cats, 330 PKJs are contained within a 1 mm^2^ sheet of cerebellar cortex arranged in a grid-like arrangement (Palkovits et al., 1971). We therefore modeled 16 PKJs along a one dimensional grid with an even 64 μm spacing between cell body centers and assume PKJ cell bodies are 32 μm in diameter. The network includes 160 MLIs in accordance with the anatomical data of a 10:1 ratio of MLIs to PKJs (Korbo et al., 1993). Thus, each PKJ has 10 nearest MLIs; three are designated as lower molecular layer interneurons and are eligible to receive PKJ recurrent collaterals (described below). Figure 1 illustrates the network and basic connectivity. Synapse formation in the network is probabilistic subject to the anatomical constraints described next.

*In vivo*, PKJ recurrent collaterals extend parasagittally and can span more than 200 μm, appearing to contact both PKJs and MLIs in the lower molecular layer (Chan-Palay, 1971; Hawkes and Leclerc, 1989; O'donoghue et al., 1989; Apps and Hawkes, 2009). Watt et al. (2009) showed PKJ recurrent collaterals extend asymmetrically and predominantly terminate within 100 μm of the parent cell soma but do not make functional synapses onto PKJs after post-natal day 21. To model this, PKJs in our network model extend their recurrent collaterals asymmetrically in the vicinity of the two nearest PKJs and can form synapses onto MLIs in the lower molecular layer, i.e., the first three of 10 MLIs corresponding to a particular PKJ. We chose a probability of forming a PKJ → MLI synapse such that, on average, each eligible MLI receives one PKJ input and each PKJ forms synapses onto three MLIs. Table 1 summarizes these convergence and divergence values. We assume the model network belongs to an adult animal and do not allow PKJ to PKJ connections.

**Table 1:** **A summary of the neuron model and network parameters**.

**Cell parameters**	**Neuron type**	
	**PKJ**	**MLI**
V_threshold_ (mV)	−55.0	−53.0
C (pF)	107.0	14.6
g_leak_ (nS)	2.32	1.6
E_leak_ (mV)	−68.0	−68.0
g_GABA_ (nS)	1.0	4.0
E_GABA_ (mV)	−75.0	−82.0
τ_GABA_ (ms)	10.0	4.6
g_AHP_ (nS)	100.0	50.0
E_AHP_ (mV)	−70.0	−82.0
τ_AHP_ (ms)	2.5	2.5
κ	0.430303	3.966333
β	0.195962	0.006653
MLI convergence	20	4
PKJ convergence	–	0.3^*^
MLI divergence	2	4
PKJ divergence	–	3

*PKJ, Purkinje cell (Puia et al., 1994; De Schutter and Bower, 1994a); MLI, molecular layer interneuron (Midtgaard, 1992; Hausser and Clark, 1997; Kondo and Marty, 1998; Lachamp et al., 2009). –, Non-existent. Convergence and divergence values are averages since the network is constructed randomly subject to anatomical constraints*.

* *Convergence was calculated on average for the entire population of MLIs, despite PKJ → MLI synapses only being made on the lower molecular layer interneurons*.

*In vivo*, MLI axons extend parasagittally and terminate up to 500 μm away from the parent soma, contacting both PKJs and other MLIs (Itō, 1984). In the model, we assume MLI axons extend asymmetrically and span a distance of eight PKJs. Each MLI axon branches in one direction or the other determined randomly with equal probability; i.e., an MLI can form synapses with MLIs and PKJs either to its left or to its right, but not both directions. We chose a probability of forming MLI → PKJ synapses such that, on average, 20 MLIs formed synapses onto one PKJ, consistent with the anatomical data (Eccles et al., 1967; Palay and Chan-Palay, 1974). Synapse formation is determined by iterating through the list of candidate target neurons for a source neuron and randomly drawing a value from *Unif(0, 1)*; if the drawn value is less than some chosen probability, then a synapse is formed. Thus, all target neurons within the axon span had an equal probability of forming a synapse whereas those neurons outside this distance had zero probability of forming synapses. We also chose a probability of forming MLI → MLI synapses such that, on average, each MLI received inputs from four other MLIs, consistent with physiological data (Hausser and Clark, 1997; Kondo and Marty, 1998). While gap junctions between MLIs are known to exist (Mann-Metzer and Yarom, 1999), we chose to model only chemical synapses as a first approximation to this network.

Peak inhibitory post synaptic conductances (IPSCs) for each neuron type are summarized in Table 1. These peak IPSCs are multiplied by synaptic weights, specific to each synapse (Equation 2). Synaptic weights are drawn from a random distribution to simulate the diversity of synaptic conductances up to the peak conductance as observed *in vitro* (e.g., Kondo and Marty, 1998). MLI → MLI synapse weights are drawn from a uniform distribution between 0 and 1, i.e., *w_MLI → MLI_* ~ *Unif*(0, 1); also, *w_MLI → PKJ_* ~ *Unif*(0, 1.25) and *w_PKJ → MLI_* ~ *Unif*(0, 1).

### Neuron model

Neurons are modeled as conductance-based leaky integrate-and-fire units (Gerstner and Kistler, 2002). The membrane potential, *V(t)*, is governed by Equation (1), where *C* is the membrane capacitance, *g_leak_* is a constant leak conductance, *g_ahp_* (*t*) is an after-hyperpolarization (AHP) conductance (described by Equation 4), *g_GABA_* (*t*) is the inhibitory GABA conductance and *I_spont_* (*t*) is a spontaneous depolarizing current (described below). *E_leak_*, *E_ahp_*, *E_GABA_* are the respective reversal potentials. Table 1 summarizes the physiological values used in the neuron models derived from the literature. The model did not include any excitatory synaptic conductances.

(1) $$\begin{array}{l} C\frac{dV}{dt}=-g_{leak}\left((V(t)-E_{leak}\right)-g_{ahp}(t)\left(V(t)-E_{ahp}\right)\\ \;-g_{GABA}(t)\left(V(t)-E_{GABA}\right)+I_{spont}(t) \end{array}$$

The total synaptic conductance is described by Equation (2), where *g*_*GABA*_ is the maximum synaptic conductance, *w_i_* is the weight of the *i^th^* synapse, α(*t*) is the conductance kinetics function described by Equation(3), and δ_*i*_ (*t*) is a Dirac delta function for the *i^th^* synapse onto a target neuron, indicating whether the presynaptic neuron has spiked at time *t*. τ_*GABA*_ is the inhibitory conductance time constant.

(2) $$g_{GABA}(t)={\bar{g}}_{GABA}\sum_{i}w_{i}\int_{-\infty }^{t}\alpha (t-s)\delta_{i}(s)ds$$

(3) $$\alpha (t)=\exp\left(-t/\tau_{GABA}\right)$$

When the membrane potential for the neuron model surpasses *V_threshold_*, the neuron emits a spike and an AHP conductance is triggered. The AHP is described by Equation (4), where *t_spiked_* is the time the neuron last spiked and τ_*ahp*_ is a time constant.

(4) $$g_{ahp}\left(t-t_{spiked}\right)=\exp\left(\frac{-(t-t_{spiked})}{\tau_{ahp}}\right)$$

**Figure 2:**
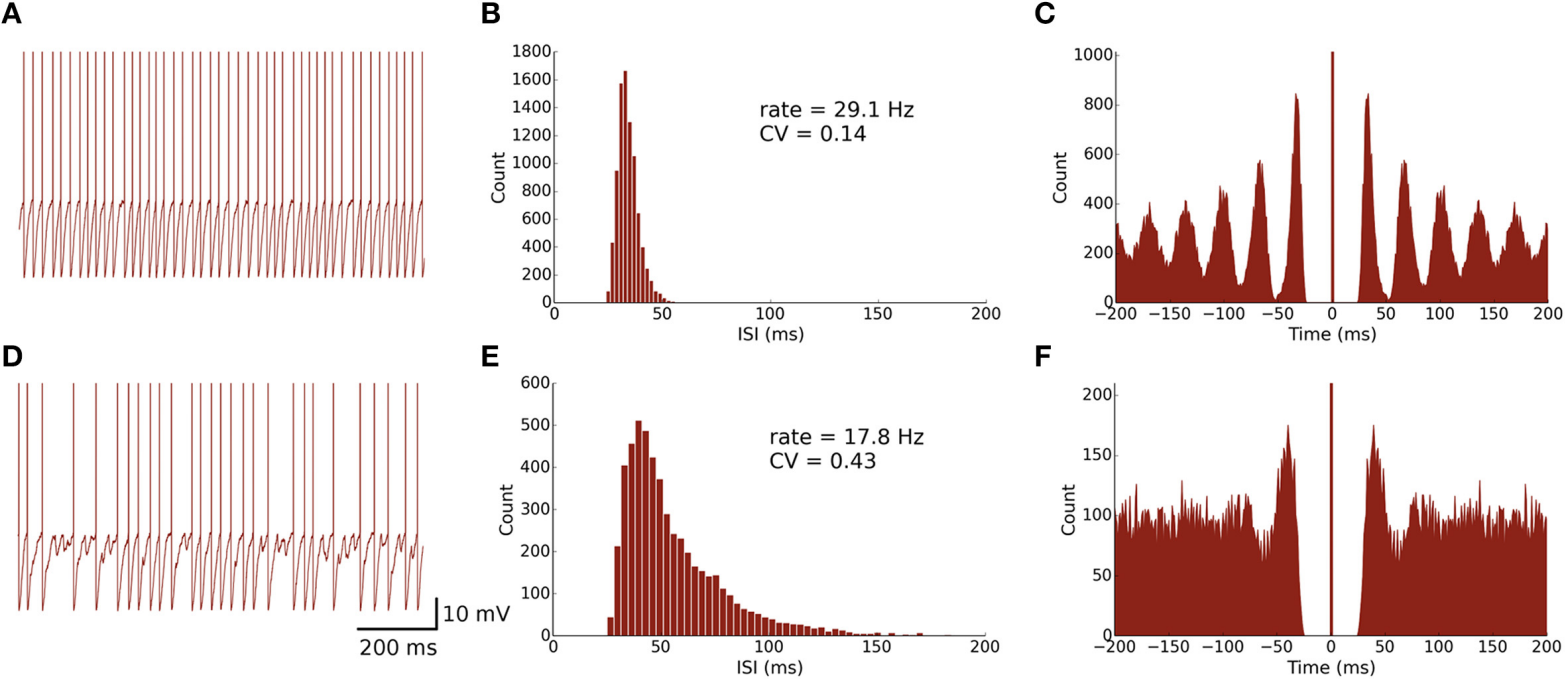
**Spontaneously active MLI neuron models reproduce similar firing patterns as observed *in vitro*. (A)** Trace of an isolated MLI membrane potential with spikes artificially drawn. The neuron appears to fire regularly in absence of inhibitory synaptic currents. **(B)** Inter-spike interval (ISI) histogram of the isolated MLI. The parameters for the gamma distribution governing the random depolarizing current injected into the neuron were chosen such that the mean firing rate and ISI coefficient of variation (CV) were similar to the example neuron shown in Hausser and Clark (1997). All simulations were run for 300 s. **(C)** A spike autocorrelogram of the isolated MLI showing regularity in trains of spikes. **(D)** Membrane potential trace of one MLI selected from the intact network of MLIs and PKJs where inhibitory synaptic currents a present. From the trace, the neuron visibly fires irregularly compared to the isolated case. **(E)** An inter-spike interval histogram of the same MLI. The distribution shifts rightward and becomes broader, suggesting a slower and more irregular firing pattern. **(F)** A spike autocorrelogram of the same MLI showing the regularity in spike trains has disappeared.

**Figure 3:**
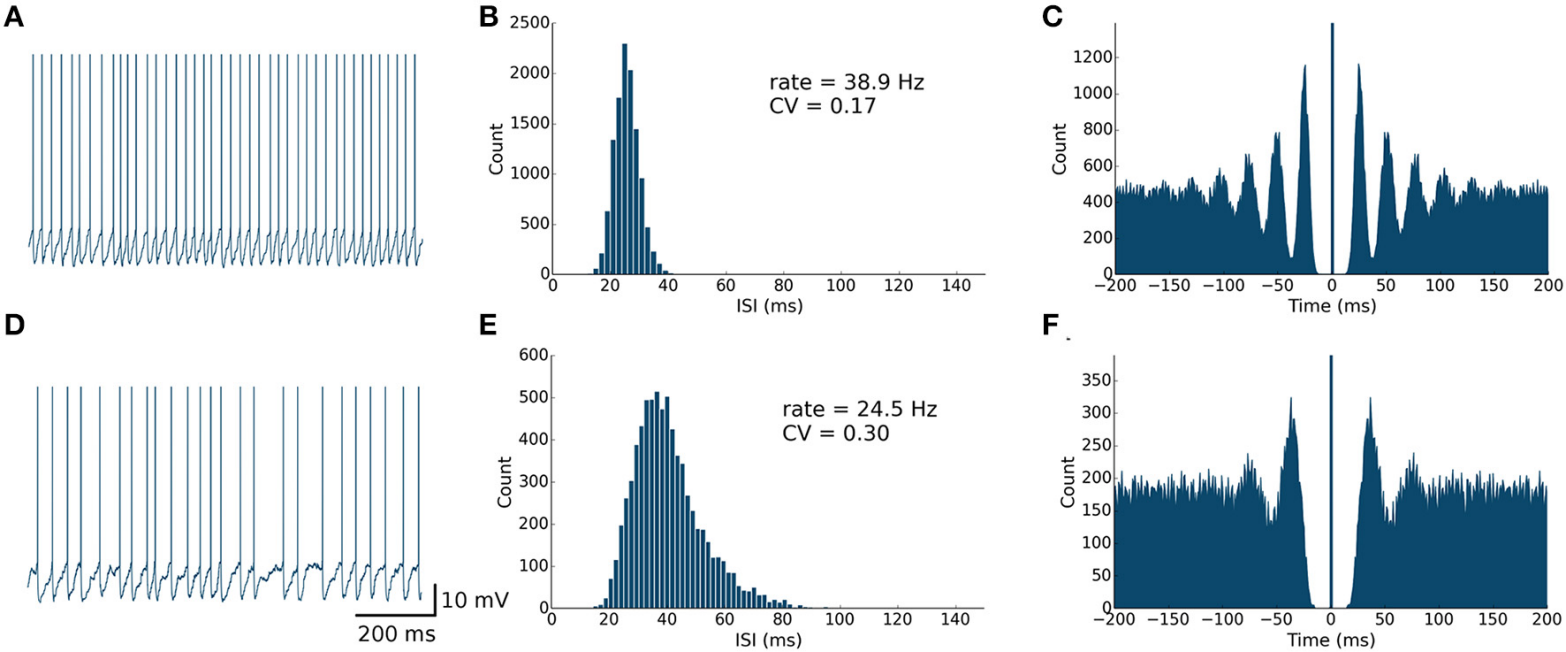
**Spontaneously active PKJ neuron models reproduce similar firing dynamics as observed *in vitro***. Conventions are as in Figure 2.

The spontaneous firing activity of MLIs and PKJs has been shown to be an intrinsic neuron property and not driven by the background activity of parallel fibers (Hausser and Clark, 1997). In PKJs, the spontaneous firing is primarily mediated by tetrodotoxin (TTX) sensitive sodium channels which produce a sub-threshold depolarizing current (Raman and Bean, 1999). While the mechanism for this endogenous current in MLIs is not well-studied, MLIs presumably share a similar mechanism to PKJs since blocking TTX-sensitive sodium channels abolishes this sub-threshold depolarizing response in MLIs (Midtgaard, 1992). To model this spontaneous activity of MLIs and PKJs, we inject a random depolarizing current drawn from a gamma distribution, *I_spont_* (*t*) ~ Γ (κ, β) (in units of nA), every time step of the simulation. A gamma distribution was chosen since its support is strictly non-negative and has flexible shape and scale (controlled by κ and β, respectively). We performed a grid search over κ and β for MLIs and PKJs separately to find the parameters which resulted in the neuron model reproducing the mean firing rate and inter-spike interval coefficient of variation (CV) that was close to the example data reported in Hausser and Clark (1997) in the presence of GABA blockers. Table 1 summarizes gamma distribution parameters for each neuron type. Figures 2, 3A–C show the resulting neuron model activity when neurons are isolated, i.e., no synaptic inputs are present.

We used the PKJ model parameters from Yamazaki and Nagao (2012) but replaced the constant spontaneous current with one drawn from a gamma distribution. A single neuron model for basket and stellate cells was derived from physiological data reported in the literature (Table 1). Anatomical and physiological evidence suggests that basket and stellate cells belong to one homogenous group of interneurons whose properties vary smoothly by depth of the soma in the molecular layer (Sultan and Bower, 1998; Ruigrok et al., 2011; Chu et al., 2012) and which share common receptive field properties (Jorntell and Ekerot, 2003). Since PKJs and MLIs are modeled as single compartment neuron models, we combine the effects of stellate-type synapses onto the PKJ dendrites with basket-type synapses onto the PKJ somas (Eccles et al., 1967; Palay and Chan-Palay, 1974) by modeling a single idealized MLI that makes synapses of one type onto the model PKJ.

### Software and data analysis

Simulations were performed using BRIAN Simulator, a Python library for spiking neural network simulations (Goodman and Brette, 2009). Simulations were carried out using Euler's method for temporal integration with a time step of 0.25 ms for numerical stability. Data analysis and plotting were performed using BRIAN Simulator, SciPy, Matplotlib, Plotly and custom software written in Python. The source code is freely available online at: http://dx.doi.org/10.5281/zenodo.12798.

Artificial action potential (AP) waveforms were drawn for Figures 2A,D, 3A,D, **5A,B**. For Figures 2, 3, a value of 0 mV was inserted when the spike occurred. For **Figure 5**, these waveforms were a hand-crafted series of six values at 0.25 ms intervals for a total AP waveform length of 1.5 ms. Exact values can be found in the published code. Mean, variances and coefficient of variations were computed assuming a normal distribution in all cases to make the values comparable to Hausser and Clark (1997).

## Results

### Model PKJs and MLIs in isolation exhibit regular firing

First, we examined the spike patterns of isolated MLI and PKJ neuron models (no synaptic currents) with spontaneous depolarizing currents. The top rows of Figures 2, 3 show the response of a model MLI and PKJ, respectively, under these conditions. The random current was sufficient to drive the neuron past threshold potential to fire spontaneously (Figures 2, 3A). A histogram of the inter-spike intervals (ISIs) reveals the degree of regularity of firing by the average baseline firing rate of the neuron and variability in timing between spike pairs (Figures 2, 3B). These results are consistent with MLIs and PKJs recorded *in vitro* when GABAergic transmission has been blocked chemically (Hausser and Clark, 1997). The model PKJ produced a mean firing rate of 38.9 Hz and an ISI CV of 0.17 compared to 40 Hz and 0.18, respectively, for an exemplar neuron *in vitro* (Hausser and Clark, 1997). The model MLI produced a mean firing rate of 29.1 Hz and an ISI CV of 0.14 compared to 30 Hz and 0.14, respectively, for an exemplar neuron *in vitro* (Hausser and Clark, 1997). The model MLI appeared slightly more skewed toward longer ISIs compared to the *in vitro* data, possibly due to longer recording times of 300 s in our experiments. While the model PKJ ISI histogram appeared symmetric, it failed a test of normality (Shapiro-Wilk test, *p* < 10^−12^) as did the MLI ISI histogram (Shapiro-Wilk test, *p* < 10^−38^). Tests of normality were not reported by Hausser and Clark (1997), though the authors noted Gaussian-shaped ISI histograms. A spike autocorrelogram revealed regularity in trains of successive spikes with several peaks at integer multiples of the baseline frequency (Figures 2, 3C). These results suggest that a simple neuron model with a spontaneous random current is capable of reproducing similar spike timing phenomena as observed *in vitro* under conditions of GABAergic transmission block.

### Model PKJs and MLIs in the network exhibit irregular firing

**Figure 4:**
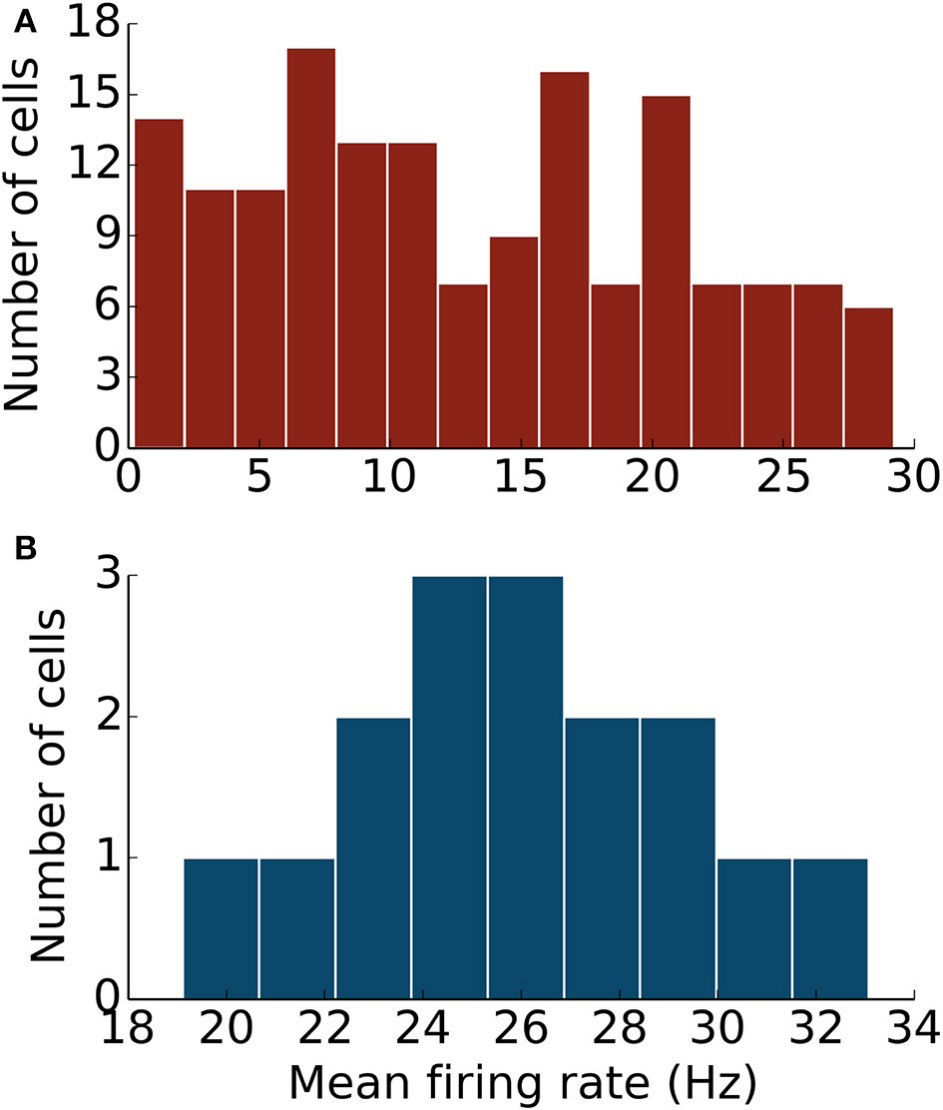
**The network of MLIs and PKJs exhibits a diversity of responses**. **(A)** A histogram of mean firing rates of MLIs from the network during one simulation. **(B)** A histogram of mean firing rates of PKJs from the network during one simulation.

Next, we examined the spike patterns of interconnected, spontaneously active MLI and PKJ neurons in a network (Figure 1). We used the same neuron models for MLI and PKJ neurons, respectively, with dynamics depicted in the top panels of Figures 2, 3, to form the network. Despite the same prototypical MLI and PKJ being used repeatedly, the random connectivity and random synaptic weight assigned when constructing the network resulted in a diversity of neuron responses (Figure 4) with MLI mean firing rates of 13.1 ± 8.0 Hz (*n* = 160, range: 0.2–29.2 Hz) and PKJ mean firing rates of 25.9 ± 3.5 Hz (*n* = 16, range: 19.1–33.1 Hz). The firing patterns of MLIs and PKJs in the network changed substantially due to the constant bombardment by inhibitory postsynaptic currents (IPSCs) from presynaptic neurons. The decreased firing rate and irregular spiking of these neurons is apparent in a trace of the membrane potential (Figures 2, 3D). The ISI histogram becomes significantly skewed favoring longer and more irregular ISIs (Figures 2, 3E). MLI ISI coefficients of variation increased markedly from the isolated case to 0.61 ± 0.24 (range: 0.14–1.04; *n* = 160), as did the PKJ ISI CVs 0.28 ± 0.04 (range: 0.21–0.39; *n* = 16). Hausser and Clark (1997) reported ISI coefficients in control conditions of 0.51 ± 0.024 (range: 0.19–0.85; *n* = 43) for MLIs and 0.28 ± 0.038 (*n* = 160, range: 0.05–1.13; *n* = 68) for PKJs. We also found examples of both MLIs and PKJs in the model network that closely matched exemplar neurons reported *in vitro* data in control conditions. A model PKJ found in the network produces a mean firing rate of 24.5 Hz and an ISI CV of 0.30 compared to 35 Hz and 0.49, respectively, for an exemplar neuron *in vitro* (Hausser and Clark, 1997). A model MLI found in the network produces a mean firing rate of 17.8 Hz and an ISI CV of 0.43 compared to 15 Hz and 0.40, respectively, for an exemplar neuron *in vitro* (Hausser and Clark, 1997). It should be noted that the background activity of parallel fiber input is present in control conditions reported for *in vitro* data but was shown to contribute only a modest increase in MLI and PKJ firing rates in a separate experiment of the same study. No parallel fiber background activity is present in this model. A significant correlation between mean firing rate and CV was found in both MLIs (Spearman rank-order coefficient *r* = −0.996, *p* < 10^−167^) and PKJs (Spearman rank-order coefficient *r* = −0.991, *p* < 10^−12^). Many of the peaks in the spike autocorrelogram disappeared suggesting that trains of spikes are no longer regularly spaced. These results suggest that a simple neuron model of spontaneously active MLIs and PKJs when interconnected in accordance with known anatomy is capable of reproducing the irregular firing patterns of MLIs and PKJs observed *in vitro*. Finally, analyses of the consistency of the model results among different random instantiations of the network constrained by the same parameters and of the robustness of the model to random perturbations in the parameters of up to 10% of the original values were performed (Figures S1–S3). These results suggest the model reproduces similar results under different random instantiations of the network (Figures S1, S2) and is robust to small changes in the parameters (Figure S3).

### Feedforward inhibition produces variable delays in the postsynaptic neuron

**Figure 5:**
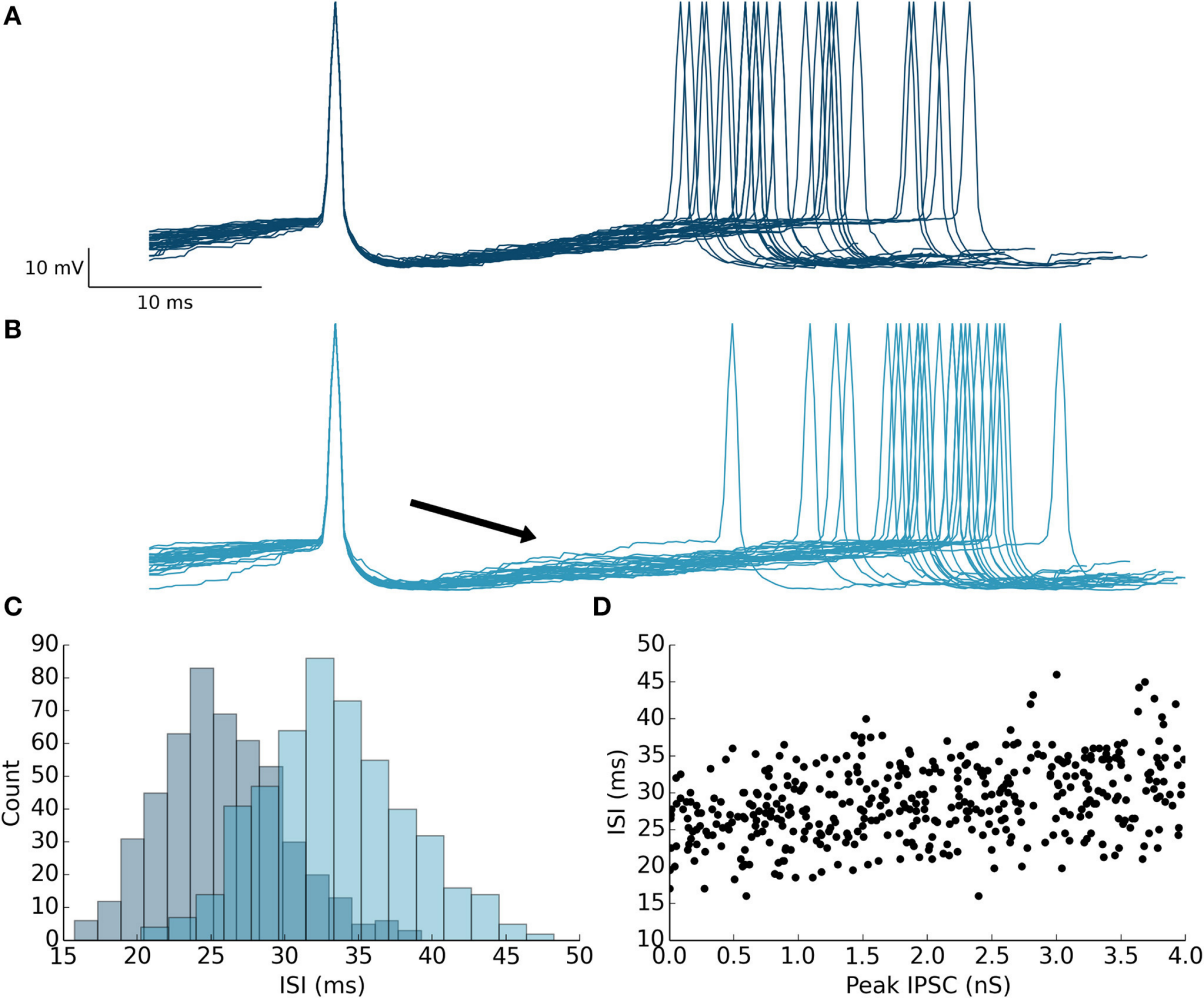
**Feedforward inhibition causes prolonged inter-spike intervals in the target neuron**. **(A)** 30 membrane potential traces overlaid with first spike aligned from a single isolated PKJ. Spikes are artificially drawn. The variable ISI can be seen. **(B)** A model MLI was then synaptically connected to the PKJ providing feedforward inhibition and caused to fire 12 ms (marked by the black arrowhead) after the first PKJ spike. 30 membrane potential traces with the first spike aligned from the PKJ are shown. The effect of the IPSC (4 nS peak in this simulation) can be shown to increase the average ISI. **(C)** Histograms of ISIs in the case without feedforward inhibition (as in **A**) (darker shade, left histogram) and with feedforward inhibition (as in **B**) (lighter shade, right histogram). **(D)** The relationship between IPSC and ISI can be seen by varying the synaptic conductance randomly in separate trials and measuring the resulting ISI.

Next, we ran simulations to illustrate the effect of feedforward inhibition on the membrane potential of PKJs between successive spikes. Multiple traces of the membrane potential of an isolated PKJ showing two successive spikes are aligned to the first spike and overlaid (Figure 5A). Action potential waveforms have been artificially drawn since the leaky integrate-and-fire model does not explicitly model the membrane potential during action potentials. The random spontaneous current resulted in variable delays between spikes. A model MLI was then synaptically connected providing feedforward inhibition onto the PKJ with a peak IPSC of 4 nS. The MLI was triggered to fire 12 ms after the PKJ's first spike. The effect of feedforward inhibition from the MLI to the PKJ delays the time of the second PKJ spike (Figure 5B). The mean delay with feedforward inhibition is significant (Mann–Whitney *U*-test, *p* < 10^−96^, *n* = 500) (Figure 5C). Moreover, a linear relationship between the peak IPSC and the ISI can be seen (Figure 5D). This suggests the mean ISI is a function of the total synaptic conductance during the interval preceding the second spike. More elaborate methods for characterizing the response of neurons to synaptic input, such as measuring the phase response curves (PRC) of PKJs (Phoka et al., 2010), can be straightforwardly applied to this model in future work.

### The effects of removing MLI → MLI or PKJ → MLI synapses

**Figure 6:**
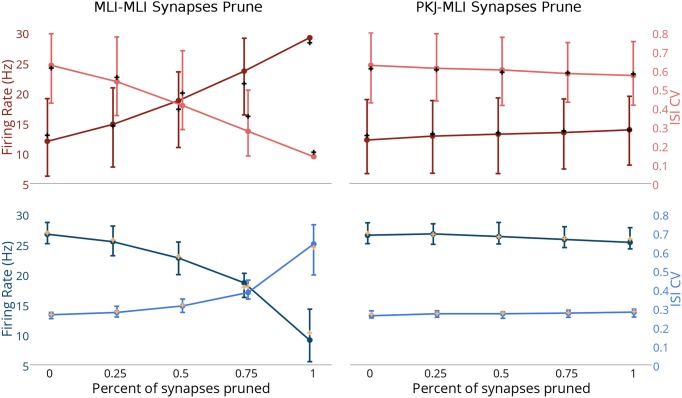
**Changes in network activity by pruning MLI → MLI or PKJ → MLI synapses**. Simulations were performed where a random set of synapses of either MLI → MLI or PKJ → MLI connections were removed from the model network to investigate the effect of these connection types on the activity of the network. Left column: measurements of MLI population (top row, in red) and PKJ population (bottom row, in blue) firing rates (darker shade) and ISI CVs (lighter shade) when MLI → MLI synapses are randomly pruned by 25, 50, and 75% as well as fully intact (0%) and fully pruned (100%, i.e., no MLI → MLI left synapses at all). Each neuron's mean firing rate and ISI CV was measured over a 60 s simulation of the operation of the network. Solid circles denote the median of the population for each of these statistics. Bars show the first and third quartile to depict the distribution of values across the population. The cross mark denotes the population mean. Right column: similar measurements in the case of PKJ → MLI synapses pruned. Top-left panel: As more MLI → MLI synapses are pruned, the median firing rate of the population of MLIs (dark red) increases due to decreased mutual inhibition. When the synapses are completely pruned, there is very little variance in the population response and quartile bars overlap with the filled circle and are not visible. Additionally, the median ISI CV decreases as more MLI → MLI synapses are pruned (light pink). Bottom-left: As more MLI → MLI synapses are pruned, inhibition onto PKJs from MLIs increases, thus decreasing the median PKJ population firing rate (dark blue) and increases the median PKJ ISI CV (light blue). Top-right: pruning PKJ → MLI synapses has only a subtle effect on the MLI population median firing right and ISI CV. Bottom right: similarly, pruning PKJ → MLI synapses has only a subtle effect on the PKJ population median firing right and ISI CV.

Finally, to explore the effects of MLI → MLI and PKJ → MLI connections on the baseline activity of the network, we simulated the network activity when a random subset of synapses from one connection type or the other were randomly pruned (i.e., removed). We simulated scenarios where a random subset of 25, 50, or 75% of the original MLI → MLI or PKJ → MLI synapses were pruned, as well as when the network is fully intact (0%) or all synapses of that connection type are removed (100%) (Figure 6). The activity of each neuron was recorded for 60 s and a mean firing rate and mean ISI CV were calculated for each neuron in each neuron-type population. The median (i.e., second quartile) of each population for both measures was computed and is depicted with filled circles. The first and third quartiles for each measure was also computed and is depicted with bars—where the lower bar is the first quartile and the upper bar is the third quartile—to show the distribution of values across the population. The population mean was also computed and is depicted by a cross in a contrasting color. As more MLI → MLI synapses are pruned, the MLI firing rates (Figure 6, top-left panel, dark red line) increase due to decreased mutual inhibition. The MLI ISI CVs (light red) decrease due to increased regularity in firing. The result of increased MLI firing is increased inhibition onto PKJs, resulting in decreased PKJ firing rates (Figure 6, lower-left panel) and increased PKJ ISI CVs. In contrast to the significant changes in MLI and PKJ firing rates and ISI CVs when MLI → MLI synapses are pruned, pruning PKJ → MLI synapses has only a subtle effect on the activity of MLIs (Figure 6, top-right) and PKJs (Figure 6, bottom-right). Statistical tests show that the difference between population firing rates in the fully intact network (0%) and the fully pruned PKJ → MLI connections (100%) is not significant for MLIs (Mann–Whitney *U*-test, *p* > 0.13, *n* = 160) or PKJs (Mann–Whitney *U*-test, *p* > 0.19, *n* = 16). These results show that MLI → MLI mutual inhibition has a significant influence on the baseline activity of the network by governing the average firing rate and variability of spike timing of MLIs and PKJs whereas the effect of PKJ → MLI connections on the baseline activity of the network is more subtle.

## Discussion

In this study, we demonstrate that a network composed of simple neuron models of MLIs and PKJs is sufficient to reproduce the irregular firing patterns of their biological counterparts as observed *in vitro*. The key elements to the model are neurons with endogenous depolarizing currents that are interconnected via inhibitory synapses in accordance with known anatomy. The random endogenous current drives each neuron to spike in the absence of all input to the neuron in a regular but still variable way. In the event of an inhibitory input from another neuron, the membrane potential of the target neuron is temporarily decreased, requiring more spontaneous depolarizing current and thereby more time to reach threshold, resulting in a longer inter-spike interval. The time between two spikes is dependent on the amount of the endogenous current and the amount of inhibitory post synaptic conductance. The results suggest that a more elaborate neuron model is not necessary to reproduce these phenomena. In addition, simulations investigating the relative importance of MLI → MLI and PKJ → MLI connections on regulating the baseline activity of the network revealed the significant role of MLI mutual inhibition to achieve results matching *in vitro* data and relatively subtle role of MLI → PKJ synapses. Finally, this network model provides a substrate for additional experimental investigation into the role of MLIs in cerebellar learning and function.

### Implications of irregular firing

Whether irregular firing has a functional role or is simply a consequence of interconnected spontaneously active neurons is not clear. Some evidence suggests a functional role for these firing patterns. Wulff et al. (2009) found that genetically modified mice lacking PKJ GABA_A_ receptors exhibited normal motor performance but were unable to consolidate motor learning following VOR gain down training. Interestingly, while the ISIs of PKJs were more regular in the genetically modified mice compared to control, the mean firing rate was nearly the same. Motor learning in the cerebellum may initially take place in the cortex and then be partially transferred to the deep cerebellar nuclei/vestibular nuclei (DCN/VN) where it is consolidated for long-term storage (Kassardjian et al., 2005; Shutoh et al., 2006). While the overall quantity of PKJ inhibition onto their targets in the DCN/VN is unchanged in knockout mice, the quality of PKJ firing patterns may be enough to disrupt consolidation of memory to the DCN/VN and could explain the failure of knockout mice to consolidate VOR gain down learning. This is consistent with electrophysiological results showing that PKJ inhibition onto DCN/VN targets controls learning at mossy fiber (MF) to DCN/VN synapses (McElvain et al., 2010; Person and Raman, 2010), a putative location for memory consolidation. Mechanistically, irregular PKJ firing may favor rebound depolarizations (RDs) occurring in PKJ targets in DCN/VN (Aizenman and Linden, 1999) by providing a period of intense inhibition followed by a period of relative relief, which in turn may control learning at DCN/VN synapses (Pugh and Raman, 2008). In the absence of spontaneous feedforward inhibition provided by MLIs, the PKJs fire more regularly and prevent DCN/VN targets from firing appropriately, possibly resulting in impaired memory transfer. However, too much feedforward inhibition leads to more irregular PKJ firing (Figure 6), which might also interfere with learning or motor performance. Episodic ataxia type-2 is a condition caused by mutations to P/Q-type voltage-gated calcium channels expressed in PKJs which leads to increased irregularity in PKJ firing and impaired motor performance (Walter et al., 2006). Thus, feedforward inhibition onto PKJs must be carefully balanced to achieve stable learning and motor performance.

The irregular firing of PKJs may also be a means of preventing synchronous PKJ activity during periods of rest when the cerebellar cortex is not actively emitting control signals. If many PKJs did synchronize their firing in response to input stimulus, then the summed activity could encode a sequence of ON periods, when most PKJs are firing, and OFF periods, when most PKJs are silent. Maex and De Schutter (2003) showed computationally that the synaptic conductance delay in a homogeneous network of inhibitory neurons is the primary parameter controlling the frequency of synchronicity among these neurons. While this model does not implement spike propagation or synaptic transmission delays, this could be one way of evoking synchronized activity among MLIs and PKJs. This ON-OFF pattern might be a means of implementing Pulse Width Modulation (PWM), a digital control signal used to represent analog values. Person and Raman (2012) found that many synchronous inhibitory inputs to a neuron in the DCN/VN can entrain the neuron to fire at a high and regular rate. This firing rate could be the analog value desired by the PWM control scheme. On the other hand, if irregular firing prevents PKJ synchrony during behavior as well, then the summed activity of asynchronous PKJs could represent an analog value for control of the DCN/VN targets. It is also possible that PKJs can switch between operating modes to convey the most appropriate control signal.

### Function of MLI-PKJ network

A more general inquiry is into the functional role of spontaneously active PKJs and MLIs. One advantage of spontaneously active neurons is that their firing rates can be both increased and decreased, by excitation and inhibition, respectively. Presumably, PKJs need to actively inhibit their DCN/VN targets which are spontaneously active and exhibit rebound depolarizations (Aizenman and Linden, 1999). Tonic inhibition by PKJs can be increased and possibly synchronized by excitatory PF inputs which could hyperpolarize or entrain DCN/VN targets. A decrease in PKJ tonic activity, via MLI feedforward inhibition, disinhibits DCN/VN targets. Such a scheme would allow for several modes of control and a similar argument can be made for the spontaneously active MLIs.

A key feature present in this model is MLI → MLI and PKJ → MLI inhibition. In the model, MLI → MLI inhibition is shown to have a significant effect on regulating the baseline firing rate and spike regularity in both MLIs and PKJs (Figure 6). As discussed, a careful balance between these two properties may be needed to ensure effective motor performance and learning. The presence of MLI → MLI inhibition also theoretically allows for competition among MLIs to take place in response to PF stimuli. Electrophysiological evidence suggests an activity dependent form of learning at PF-MLI synapses may exist (Liu and Cull-Candy, 2000; Rancillac and Crepel, 2004; Smith and Otis, 2005, but see also Jorntell and Ekerot, 2002). If this is correct, a diverse set of MLI receptive fields and responses could emerge from this competition. Plasticity at MLI → PKJ synapses (Gao et al., 2012) could enable PKJs to learn the most appropriate set of inhibitory inputs to achieve the desired output response. While the anatomical data on MLI → PKJ convergence between cat and rat differs (Eccles et al., 1967; Palay and Chan-Palay, 1974), plasticity at these synapses could also tune the total inhibitory conductance onto a PKJ to ensure that the baseline PKJ activity is appropriate. In the present model, this would be achieved by altering the synaptic weights. In another computer model, PKJ → PKJ feedback inhibition enables PKJs in a network with MLIs to perform temporal integration on a time-scale of seconds (Maex and Steuber, 2013); the role of MLI → MLI and PKJ → MLI inhibition may serve a similar function. In contrast, PKJ → MLI connections appear to have only a small effect on the resting activity of the network (Figure 6). Taken together, these ideas suggest the information storage capacity and expressiveness of the PKJ-MLI network is even greater than previous theories describe (Brunel et al., 2004; Clopath et al., 2012). The model proposed here provides an initial step toward carrying out further computational investigations into these questions.

### Comparison with other models

De Schutter and Bower (1994b) model a Purkinje cell as a Hodgkin-Huxley-type, multi-compartmental model that reproduces asymmetric ISI distributions in response to PF and MLI inputs. However, in these experiments the model PKJ relies exclusively on PF inputs to drive spiking and not an endogenous depolarizing current. Further the influence of MLIs is modeled indirectly as Poisson spike trains which assumes the ISI distribution is exponential, whereas our model generates MLI spikes by simulating MLI dynamics directly and results in an appropriate ISI distribution. Finally, by simulating the network of MLIs and PKJs, our model enables simulating the response of the MLI-PKJ network to PF input to investigate cerebellar function. Previous computational network models of the cerebellum that include MLIs typically ignore a number of anatomical or physiological facts. For example, models do not include the spontaneous activity of MLIs (Yamazaki and Nagao, 2012) or MLI → MLI and PKJ → MLI connectivity (Contreras-Vidal et al., 1997; Schweighofer et al., 1998; Medina et al., 2000; Maex and Steuber, 2013). Adaptive filter models implicitly model the inhibitory effect of MLIs by allowing the PF-PKJ filter weights to be negative (Fujita, 1982; Dean et al., 2010). While our network models a parasagittal strip of cerebellar cortex, other work has modeled a medio-lateral strip to investigate the effects of spontaneously active MLIs on PKJs along a beam of PF inputs (Santamaria et al., 2007). Further effort to extend the model proposed in our study to include the medio-lateral axis would be worthwhile.

### Conflict of interest statement

The authors declare that the research was conducted in the absence of any commercial or financial relationships that could be construed as a potential conflict of interest.
