# Supplementary material for: A spiking network model of cerebellar Purkinje cells and molecular layer interneurons exhibiting irregular firing
Source: Front Comput Neurosci. 2014 Dec 1;8:157. doi: 10.3389/fncom.2014.00157 (PMC4249458; doi:10.3389/fncom.2014.00157)
Supplement: Supplementary file 1 [file DataSheet1.DOCX]

***Supplementary Material***

**A spiking network model of cerebellar Purkinje cells and molecular layer interneurons exhibiting irregular firing**

**William Lennon*^1^, Robert Hecht-Nielsen^1^ and Tadashi Yamazaki^2^**

^1^Department of Electrical and Computer Engineering, University of California, San Diego, La Jolla, CA, USA

^2^Graduate School of Informatics and Engineering, The University of Electro-Communications, Chofu, Tokyo, Japan

*** Correspondence:** William Lennon, Department of Electrical and Computer Engineering, University of California, San Diego, 9500 Gilman Drive, La Jolla, CA, 92093, USA.
wlennon@ucsd.edu

1. **Supplementary Data**

N/A

1. **Supplementary Figures and Tables**

## Supplementary Tables

N/A

## Supplementary Figures

##
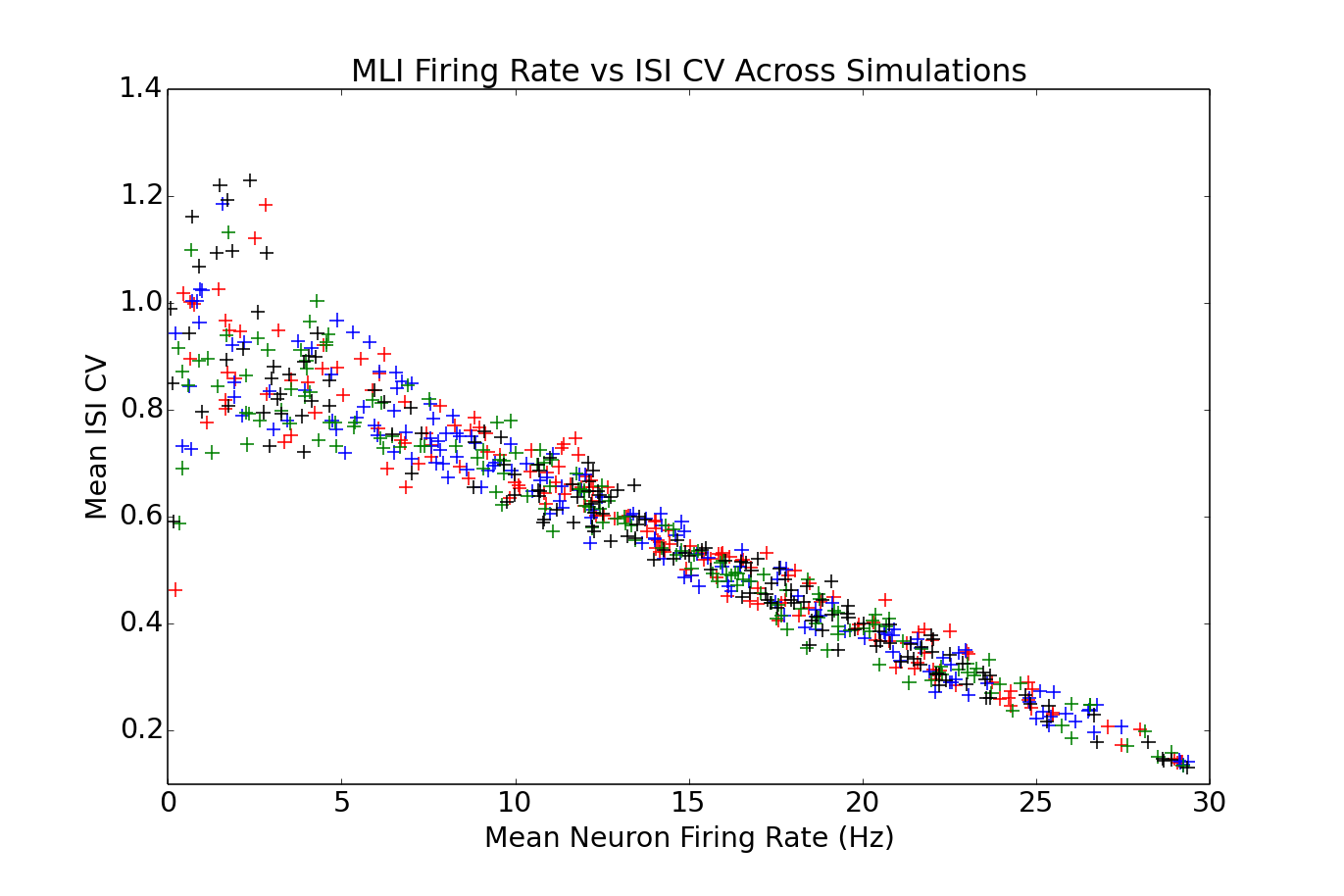


## Figure S1. Consistency analysis of the model: MLI responses. To test whether different random instantiations of the model governed by fixed parameters (synapse probabilities, axon lengths, and spontaneous current parameters) will produce consistent (similar) results, we simulated 100 different random networks constrained by the parameters described in the Methods section. From these 100 simulations, four were chosen randomly to be visualized. The mean firing rates and ISI CVs of MLIs were measured during each simulation lasting 30 seconds. A scatter plot of mean firing rate and ISI CV was plotted for each neuron (denoted with a plus sign). Each of the four randomly chosen simulation results was assigned a different color. The clouds of points generated by each simulation overlap extensively suggesting the model network consistently produces results similar to those presented in Figures 2-5 and that they are not the result of selection bias by the experimenter.


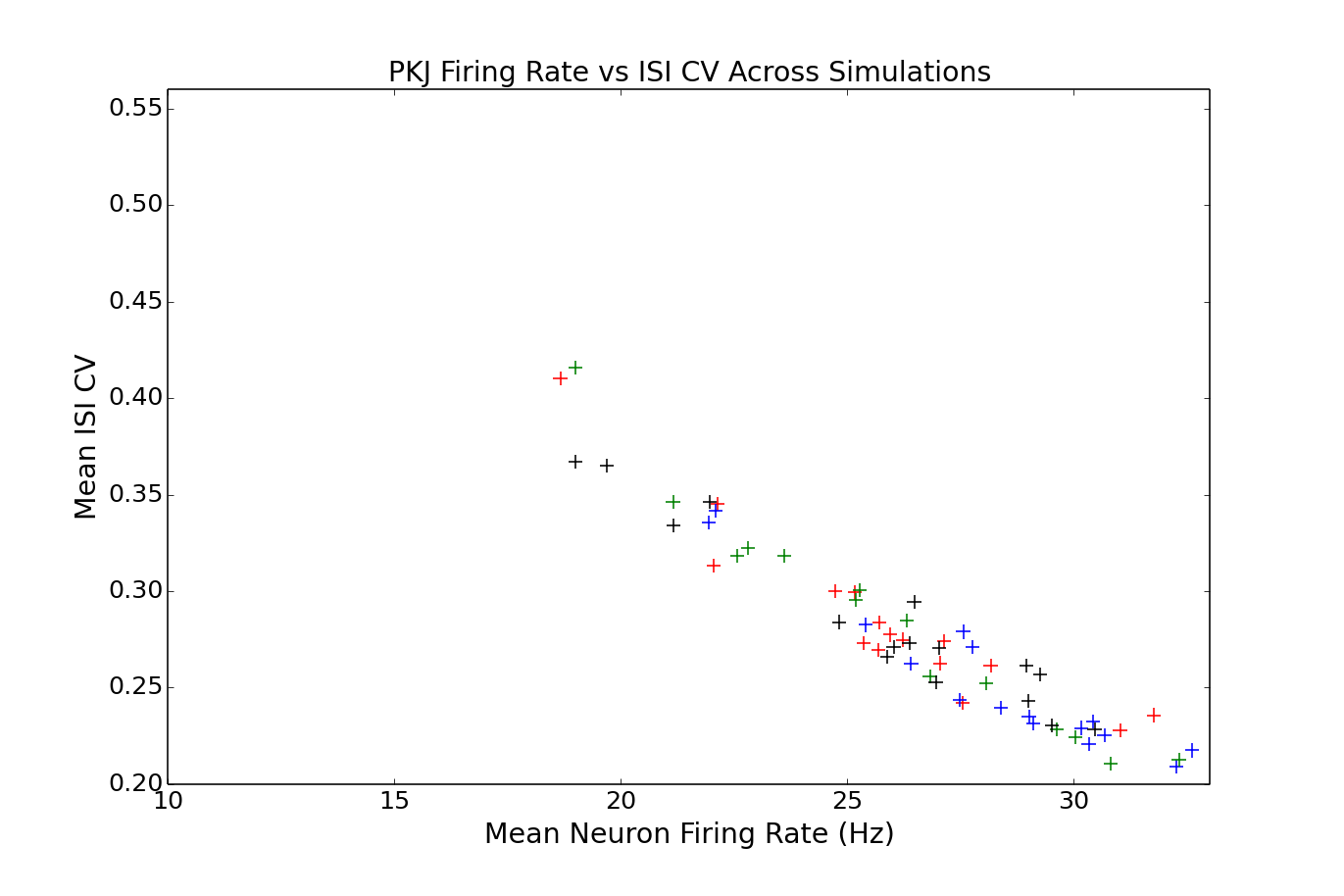


## Figure S2. Consistency analysis of the model: PKJ responses. Similar to Figure S1 but for PKJs.

##
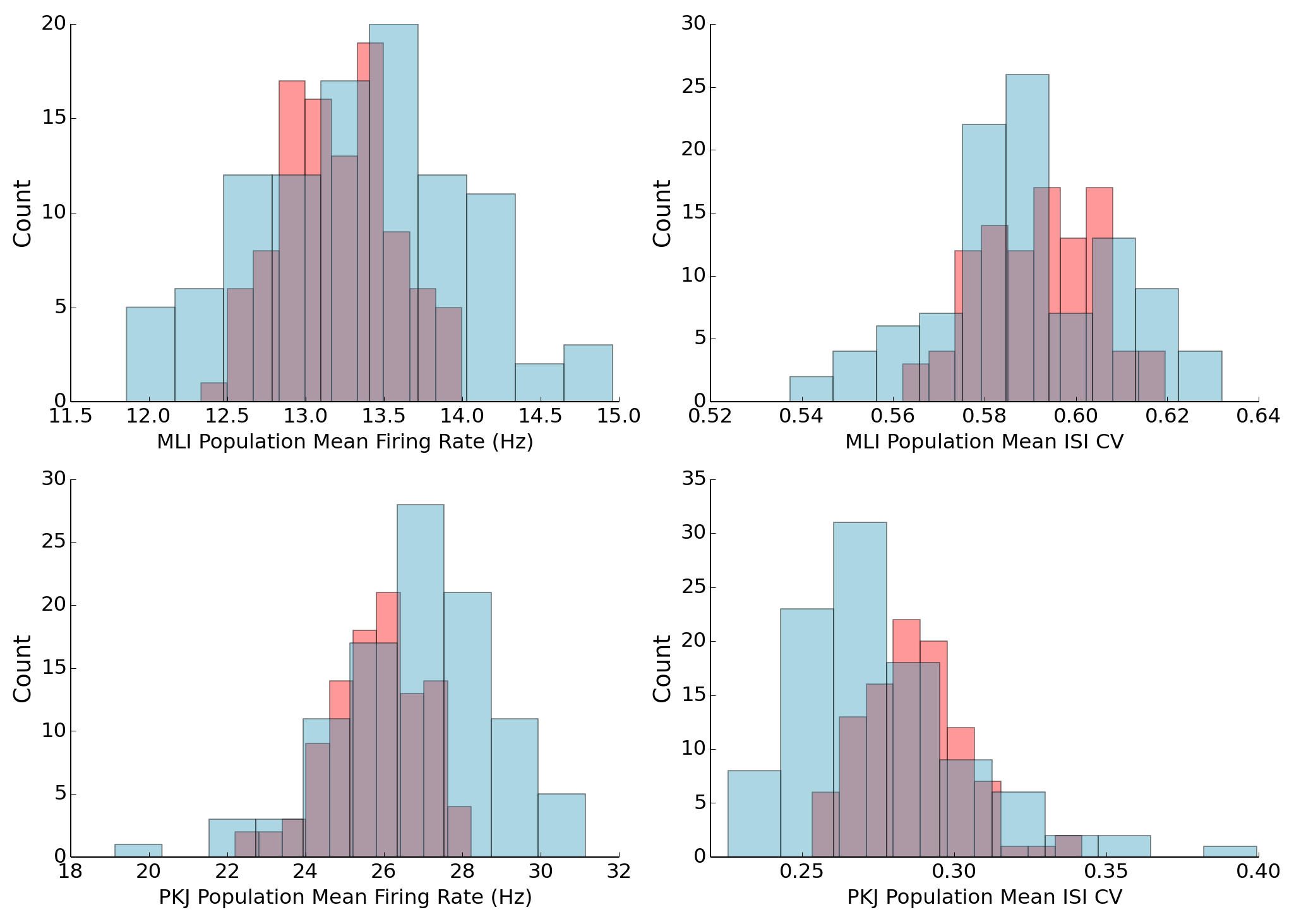


## Figure S3. Robustness analysis of the model to random perturbations in the parameters. To test the robustness of the model to changes in the parameters, 100 simulations were performed where all parameters (synapse probabilities, axon lengths, and spontaneous current parameters) were randomly perturbed by up to 10% of their original value (drawn from a uniform random distribution). Population mean firing rates and ISI CVs are computed and histograms of these values across trials are plotted. Blue histograms show the responses from the perturbed networks. Red histograms show the responses from 100 randomly instantiated networks with the original (fixed) parameters. There is significant overlap in network activity across simulations in the perturbed and unperturbed cases indicating that the network produces similar activity that is robust to small changes in the model parameters.

1. **References**

N/A
